# A multifaceted educational intervention improved anti-infectious measures but had no effect on mortality in patients with severe sepsis

**DOI:** 10.1038/s41598-022-07915-9

**Published:** 2022-03-10

**Authors:** Daniel Schwarzkopf, Claudia Tanja Matthaeus-Kraemer, Daniel O. Thomas-Rüddel, Hendrik Rüddel, Bernhard Poidinger, Friedhelm Bach, Herwig Gerlach, Matthias Gründling, Matthias Lindner, Christian Scheer, Philipp Simon, Manfred Weiss, Konrad Reinhart, Frank Bloos, MEDUSA study group, Gernot Marx, Achim Schindler, Tobias Schürholz, Heike Schlegel‑Höfner, Gunther Lehmann, Annett Sander, Steffen Friese, Christian Scholz, Pia Fischer, Christina Fuchs, Lutz Becher, Norbert Salewsky, Torsten Schreiber, Anton Goldmann, Didier Keh, Katrin Schmid, Winfried Menning, Renate Steuckart, Robert Barz, Karin Dey, Meike Fahrenholz, Martin Müller, Susanne Toussaint, Jörg Brederlau, Dirk Buschmann, Ingo Gummelt, J. Hoeschen, Marion Klaproth, Ina Vedder, Ulrike Bachmann‑Holdau, Jürgen Eiche, Rolf Hauschild, Martina Lange, Davia Herrmann‑Karbaum, Annette Lubasch, Marcus Rücker, Christian Icke, Alexander Lucht, Andreas Meier‑Hellmann, Jan Wagner, Olaf Arnold, Steffen Kästner, Tobias Clausen, Michael Sternkopf, Robert Voswinckel, T. Benndorf, Christel Eiserloh, Gerhard Kuhnle, Mathias Koch, Manuela Gerber, Liane Guderian, Sven‑Olaf Kuhn, Gerd Scheiber, Frank Bloos, Stefanie D’Aria, Thees Lemke, Birgit Michaelsen, Dirk Schädler, Nina Schulz‑Ruhtenberg, Norbert Weiler, Martin Anetseder, Zoran Textor, Udo Kaisers, Matthias Löbe, Frank Meineke, Christine Pausch, Christoph Engel, Georg Braun, Nicole Jensen, Werner Gegenfurtner, Alexander Meinhardt, Robert Schmitt, Andrea Teichert, Klaus‑Dieter Becker, Anja Diers, Florian Jelschen, Andreas Weyland, Frieder Knebel, Thomas Kupfer, Rüdinger Sinz, Petra Bautz, Annemarie Fischer, Armin Seibel, Christoph Fleischhacker, Helene Häberle, Philipp Henn, Friederike Mezger, Peter Rosenberger, Reimer Riessen, Silvia Ziegler, Eberhard Barth, Hendrik Bracht, I. Heymann, A. Hinder, R. Sens, Christof Lascho, Henriette Micke, Falk Schmidt, Stefanie Schilling, Gabriele Wöbker

**Affiliations:** 1 Integrated Research and Treatment Center-Center for Sepsis Control and Care (CSCC), Jena University Hospital, Am Klinikum 1, 07747 Jena, Germany; 2 Department of Anaesthesiology and Intensive Care Medicine, Jena University Hospital, Am Klinikum 1, 07747 Jena, Germany; 3 Center for Infectious Diseases and Infection Control, Jena University Hospital, Am Klinikum 1, 07747 Jena, Germany; 4 Department for Infectious Diseases, Protestant Hospital of Bethel Foundation University Hospital, University of Bielefeld, Bethesdaweg 10, 33617 Bielefeld, Germany; 5 Department for Anaesthesia, Intensive Care Medicine and Pain Management, Vivantes-Klinikum Neukoelln, Rudower Strasse 48, 12351 Berlin, Germany; 6 Department of Anaesthesiology, University Hospital of Greifswald, Ferdinand-Sauerbruch-Straße, 17475 Greifswald, Germany; 7 Department of Anaesthesiology and Intensive Care Medicine, University Hospital Schleswig-Holstein, Campus Kiel, Arnold-Heller-Straße 3, 24105 Kiel, Germany; 8 Department of Anaesthesiology and Intensive Care Medicine, University of Leipzig Medical Centre, Liebigstraße 20, 04103 Leipzig, Germany; 9 Klinik für Anästhesiologie und Intensivmedizin, Universitätsklinikum Ulm, Albert-Einstein-Allee 23, 89081 Ulm, Germany; 10 Department of Anaesthesiology and Operative Intensive Care Medicine (CCM, CVK), Charité Universitätsmedizin Berlin, Corporate Member of Freie Universität Berlin, Humboldt-Universität zu Berlin, Augustenburger Platz 1, 13353 Berlin, Germany; 11 Berlin Institute of Health, Campus Virchow-Klinikum, Anna-Louisa-Karsch-Straße 2, 10178 Berlin, Germany; 12 Department of Intensive Care Medicine, University Hospital RWTH Aachen, Aachen, Germany; 13 Department of Anesthesiology and Intensive Care Medicine, Ilm‑Kreis‑Kliniken Arnstadt, Arnstadt, Germany; 14 Department of Anesthesiology and Intensive Care Medicine, Helios Hospital Aue, Aue, Germany; 15 Department of Anaesthesia and Intensive Care Medicine, Zentralklinik Bad Berka GmbH, Bad Berka, Germany; 16 Department of Anesthesiology and Intensive Care Medicine, Hufeland‑Klinikum, Bad Langensalza, Germany; 17 Department of Anesthesiology and Intensive Care Medicine, Bundeswehrkrankenhaus Berlin, Berlin, Germany; 18 Department of Intensive Care Medicine, Helios Hospital Berlin‑Buch, Berlin, Germany; 19 Department of Anesthesiology, Emergency and Intensive Care Medicine, and Pain Therapy, Protestant Hospital of Bethel Foundation University Hospital, Bielefeld, Germany; 20 Department of Anesthesiology, Helios‑Hospital St. Josefs‑Hospital Bochum‑Linden, Bochum, Germany; 21 Department of Anesthesiology and Intensive Care Medicine, St. Georg Hospital Eisenach, Eisenach, Germany; 22 Department of Anesthesiology and Intensive Care Medicine, Hospital Rudolf Elle, Eisenberg, Germany; 23 Department of Interdisciplinary Intensive Care and Emergency Medicine, Helios‑Hospital Emil‑von Behring, Berlin, Germany; 24 Department of Anesthesiology and Intensive Care Medicine, Helios‑Hospital Erfurt, Erfurt, Germany; 25 Department of Anesthesiology and Intensive Care Medicine, Catholic Hospital St. Johann Nepomuk, Erfurt, Germany; 26 Department of Internal Medicine, Hospital Friedberg, Friedberg, Germany; 27 Department of Anesthesiology and Intensive Care Medicine, SRH Waldklinikum Gera, Gera, Germany; 28 Department of Anesthesiology and Intensive Care Medicine, Hospital Ilmenau, Ilmenau, Germany; 29 Department of Anesthesiology and Surgical Intensive Care Medicine, Hospital Landshut‑Achdorf, Landshut, Germany; 30 Institute of Medical Informatics, Statistics and Epidemiology, University of Leipzig, Leipzig, Germany; 31 Department of Intensive Care and Emergency Medicine, Hospital Meiningen, Meiningen, Germany; 32 Department of Anesthesiology and Intensive Care Medicine, Saale‑Unstrut‑Hospital Naumburg, Naumburg, Germany; 33 Department of Anesthesiology, Intensive Care Medicine, Emergency Medicine, and Pain Therapy, Hospital Oldenburg, Oldenburg, Germany; 34 Department of Anesthesiology and Intensive Care Medicine, Thüringen‑Klinik Pößneck, Pößneck, Germany; 35 Department of Intensive Care and Emergency Medicine, Asklepios Hospital Radeberg, Radeberg, Germany; 36 Department of Anesthesiology, Intensive Care Medicine, and Pain Therapy, Thüringen‑Kliniken Saalfeld, Saalfeld, Germany; 37 Department of Anesthesiology, Intensive Care, and Emergency Medicine, Ev. Jung‑Stilling Hospital, Siegen, Germany; 38 Department of Anesthesiology, University Hospital Tübingen, Tübingen, Germany; 39 Department of Internal Medicine, University Hospital Tübingen, Tübingen, Germany; 40 Department of Anesthesiology and Intensive Care Medicine, Hufeland Hospital Weimar, Weimar, Germany; 41 Department of Intensive Care Medicine, Helios Hospital Wuppertal, Wuppertal, Germany

**Keywords:** Infectious diseases, Outcomes research, Randomized controlled trials, Health services, Antimicrobial therapy

## Abstract

Sepsis is a major reason for preventable hospital deaths. A cluster-randomized controlled trial on an educational intervention did not show improvements of sepsis management or outcome. We now aimed to test an improved implementation strategy in a second intervention phase in which new intervention hospitals (former controls) received a multifaceted educational intervention, while controls (former intervention hospitals) only received feedback of quality indicators. Changes in outcomes from the first to the second intervention phase were compared between groups using hierarchical generalized linear models controlling for possible confounders. During the two phases, 19 control hospitals included 4050 patients with sepsis and 21 intervention hospitals included 2526 patients. 28-day mortality did not show significant changes between study phases in both groups. The proportion of patients receiving antimicrobial therapy within one hour increased in intervention hospitals, but not in control hospitals. Taking at least two sets of blood cultures increased significantly in both groups. During phase 2, intervention hospitals showed higher proportion of adequate initial antimicrobial therapy and de-escalation within 5 days. A survey among involved clinicians indicated lacking resources for quality improvement. Therefore, quality improvement programs should include all elements of sepsis guidelines and provide hospitals with sufficient resources for quality improvement.

Trial registration: ClinicalTrials.gov, NCT01187134. Registered 23 August 2010, https://www.clinicaltrials.gov/ct2/show/study/NCT01187134.

## Introduction

Sepsis is the final pathway to death from infectious diseases^1^ and affects an estimated number of 49 million patients per year worldwide of whom 11 million die^2^. Similar to stroke or myocardial infarction, sepsis needs to be treated as an emergency^3,4^. Guidelines demand early adequate anti-infectious measures—including beginning of broad-spectrum antimicrobial therapy (AT) within 1 h^4^. Adherence to guideline recommendations is associated with improved survival, but often shows as low^5–9^. Recognizing these shortcomings, the World Health Organization issued a resolution in May 2017 that urges member states to improve quality of care^10^.

Numerous studies have shown improvements in early adequate therapy of sepsis and guideline adherence as well as decreased mortality by implementing multifaceted educational interventions to improve care^11–13^. The validity of these results is questionable since the evaluation of interventions was conducted in uncontrolled before-after designs^14^. The MEDUSA trial was the first study that used a randomized controlled evaluation design. Since the intervention involved staff education and improvement of care processes throughout the participating hospitals, it could not be allocated at the individual patient level. Therefore, participating hospitals were cluster-randomized in an intervention and a control group^15^. The intervention did not result in increased guideline adherence or decreased mortality^16^. This was partly due to the fact that the intervention was not fully implemented in the participating hospitals^16,17^. Therefore, our aim was to investigate, whether a better implementation strategy of the intervention could result in improved conduction of anti-infectious measures and decreased mortality in patients with sepsis in a second phase of the trial.

## Methods

### Design and setting

The original MEDUSA study was a pragmatic, un-blinded cluster-randomized controlled trial with randomization of hospitals into one of two groups. German hospitals involved in the primary care of sepsis patients and committed to participate in a quality improvement process were invited to participate in the trial. Hospitals without an intensive care unit (ICU) were not included. Hospitals were stratified according to time to antimicrobial therapy observed in a pre-study^5^ and then randomized 1:1 to either a control group or an intervention group. Randomization was computer generated. Blinding was not possible^16^. Initially, we planned to do an evaluation phase for testing the intervention, and an implementation phase for testing the sustainability of the intervention. Since the evaluation phase (July 2011 until June 2013) did not result in marked improvements^16^, we decided to improve the intervention strategy and to test this improved intervention in the second phase of the study (September 2013 and May 2015). Former control hospitals switched to intervention while former intervention hospitals acted as controls. In the following, we refer to hospitals receiving the intervention in phase 2 as “intervention hospitals”, and to the hospitals acting as controls in phase 2 as “control hospitals”. This new study uses a difference-in-differences design by comparing the changes from phase 1 to phase 2 between groups^14^. Forty-four hospitals were randomized. The study was performed in accordance with the Declaration of Helsinki; all methods were performed in accordance with the relevant guidelines and regulations. The local ethics committees at each participating hospital approved the study. The need for informed consent was waived by the leading ethics committee—the ethics committee of the Jena University hospital (record number 2910–08/10)—since randomization was performed on the cluster level, and the interventions comprised quality improvement measures.

### Patients

All consecutive adult patients treated in the ICU for proven or suspected infection with at least one new organ dysfunction related to the infection were eligible for inclusion. Organ dysfunctions were defined according to sepsis-1 definitions, since the new sepsis-3-definitions were not published yet during conduction of the trial^18^. Patients were excluded, if a limitation of life-sustaining therapy was present at onset of sepsis (e. g. withholdings by medical decision or advance directive), and beginning of sepsis therapy in another hospital.

### Intervention

Intervention hospitals received a multifaceted intervention strategy aiming in particular at improving sepsis recognition by the medical staff and shortening time to anti-infective therapy and source control throughout the whole hospital. Within each hospital, a senior intensive care physician was responsible to guide the quality improvement (QI) efforts. The strategy consisted of (a) formation of inter-professional local QI teams including ICU and non-ICU staff, (b) educational outreach to QI teams by the study coordinators, (c) audit and feedback of quality indicators of anti-infectious measures (e.g. time to beginning of AT) by quarterly quality reports, (d) active reminders by monthly feedback of cases receiving delayed or inappropriate anti-infective treatment, and (e) passive reminders to create and maintain awareness of the project (pocket cards, flyers, posters to be distributed among hospital staff). The QI teams were encouraged to implement education on early recognition and adequate treatment of sepsis among medical staff and changes to care processes throughout the hospital. Concrete goals and QI activities were discussed during the educational outreach sessions between QI teams and study coordinators.

Based on systematic qualitative analyses of experiences of the study coordinators as well as local QI team leaders during phase 1^17^, the implementation strategy was improved for phase 2. Improvements included extending the set of measures used for audit and feedback as well as active reminders, increased efforts to include departments outside the ICU in the QI teams, improvement of structure and documentation of educational outreach sessions, conduction of focus group interviews with clinical staff on barriers to early detection and treatment of sepsis^19^. Additionally, greater emphasis was placed on appropriateness of antimicrobial therapy by extending the quality reporting and focusing this issue in educational outreach. The control condition in phase 1 consisted of standard lectures about sepsis care twice a year and regular newsletters with current sepsis-related publications or conference proceedings. During phase 2, former intervention hospitals (now controls) received quality reports (audit and feedback, active reminders) but no further guidance by the study coordinators. A detailed description of the original and improved intervention strategy is provided in the Supplementary Methods.

### Data collection

Trained research nurses or physicians in the participating centers abstracted the study data from the medical records of included patients. Although patients were included on the ICU, all available information was used—including retrospective information from patient charts of the general wards and emergency department. Time of first infection-related organ dysfunction as documented in the patient record was used to define onset of severe sepsis or septic shock. Septic shock was defined by documented vasopressor use within 12 h after sepsis onset. All-cause mortality within 28 days after onset of severe sepsis or septic shock was assessed by telephone contact if the patient was no longer in the hospital. We used the clinical data management software OpenClinica^®^ (OpenClinica, LLC, Waltham, MA, USA) for collecting the data. Data integrity was confirmed by source data monitoring of a random sample of 10% of the patients and by data checks within the database during phase 1.

#### Process of care and outcome measures

Primary outcome for the trial was 28-day mortality. Indicators of guideline adherence of care were chosen as secondary endpoints^3,4^ and included (a) the initiation of antimicrobial therapy within 1 h after sepsis onset, (b) conduction of surgical source control within 6 h after sepsis onset, (c) taking at least two sets of blood cultures, (d) acquisition of blood cultures before antimicrobial therapy, (e) appropriateness of initial antimicrobial therapy, and (f) de-escalation of antimicrobials within 5 days. Time to antimicrobial therapy is reported as the difference between onset of sepsis and first antibiotic administration, its value being negative if antimicrobial therapy was prescribed up to 24 h before the onset. Time to surgical source control was defined by the same method. Initial treatment was classified as appropriate when at least one of the drugs administered was classified as effective based on the susceptibility in the antibiogram of the causative microorganism isolated in blood cultures or other microbiological samples. When no cultures were taken or results were negative, appropriateness of treatment was classified as undetermined. Change in antimicrobial therapy during the first 5 days was classified as no change, de-escalation (less broad-spectrum coverage), escalation due to clinical deterioration, due to resistance pattern, and calculated escalation without clinical deterioration.

### Process evaluation

Since a complex intervention was conducted, it was accompanied by a process evaluation^20,21^. During phase 1 of the trial, we had conducted qualitative interviews of local QI team leaders to assess facilitators and barriers to a successful implementation^17^. To include viewpoints from more involved stakeholders, the process evaluation of phase 2 used standardized surveys. Study coordinators were surveyed after each educational outreach session with QI teams, and QI team members of the intervention group were surveyed one time during the second half of the implementation phase. The questionnaires contained open-ended questions concerning items discussed and aims set for QI during the educational sessions, and perceived barriers to implementing change. Written comments were qualitatively analyzed by content analyses and obtained categories were counted^22^.

### Statistical analysis

We used standard descriptive statistics to compare baseline characteristics and outcomes between study arms across the two phases. The statistical effect of belonging to the intervention group on patient characteristics and outcomes was tested by a difference-in-differences analysis^14^. This analysis tests if a change in a variable across phases differs between groups. Regression analyses with an interaction term between phase and group were used to test the difference-in-differences. To adjust for possible confounding, age, sex, source and origin of infection, location of the patient at sepsis onset, and vasopressor use during the first 12 h were included as covariates. To control for the clustering of cases in hospitals, the regression analyses were conducted by hierarchical generalized linear models with a random slope^23^. The link-function was chosen based on the type of variable: linear-link for continuous variables, logit-link for dichotomous variables, multinomial model with logit-link for categorical variables. Since items on appropriateness of initial therapy and change of antimicrobial therapy were newly defined in phase 2, we tested the endpoints appropriateness of initial antimicrobial therapy and de-escalation within 5 days by comparing control and intervention group during phase 2 by hierarchical generalized linear models with a logit-link and adjusted for confounders. Since selective drop-out of hospitals might have biased results, we repeated the tests on outcomes using only data of hospitals that continued to include patients until the second half of phase 2 of the trial (after 2014-07-15). For each outcome, intracluster correlation was assessed by the intraclass correlation (ICC). Tests were conducted at significance level of α ≤ 0.05. Missing data were treated by case-wise deletion. Analyses were conducted using R (Version 4.1.2; R Core Team, Wien, Austria) and SAS software, version 9.4 of the SAS System for Windows (Copyright © 2002–2012 SAS Institute Inc. SAS and all other SAS Institute Inc. product or service names are registered trademarks or trademarks of SAS Institute Inc., Cary, NC, USA). Hierarchical generalized linear models with linear-link and logit-link were fit by the functions *lmer* and *glmer* of the R-package lme4^24^, multinomial hierarchical generalized linear models were fit by the GLIMMIX-procedure of SAS.

### Ethics declaration

The study was performed in accordance with the Declaration of Helsinki, all methods were performed in accordance with the relevant guidelines and regulations. The study was approved by the local ethics committees at each participating hospital. The need for informed consent was waived since randomization was performed on the cluster level, and the interventions comprised quality improvement measures. Involved ethical bodies: Ethics committee of the University Hospital Jena (2910-08/10); Ethics committee of the “Ärztekammer Nordrhein” (2010403); Ethics committee of the “Ärztekammer Westfalen-Lippe” (2010-518-b-S); Ethics committee of the “Landesärztekammer Baden-Würtemberg” (B-F-2010-056); Ethics committee of the “Landesärztekammer Bayern” (7/10284); Ethics committee of the “Landesärztekammer Hessen” (MC 245/2010); Ethics committee of the “Landesärztekammer Niedersachsen” (Ar/211/2010); Ethics committee of the “Landesärztekammer Saarland” (195/10); Ethics committee of the “Landesärztekammer Sachsen” (EK-BR-51/10-1); Ethics committee of the Medical Faculty Leipzig (324-10-08112010); Ethics committee of the “Landesärztekammer Sachsen-Anhalt” (33/10); Ethics committee of the “Landesärztekammer Thüringen” (38831/2010/109); Ethics committee of the Medical Faculty Greifswald (BB 129/10); Ethics committee of the Medical Faculty Kiel (B 204/11); Ethics committee of the Medical Faculty Tübingen (556/2010BO2); Ethics committee of the University Ulm (295/10); Ethics committee of the University Witten-Herdecke (90/2010).

## Results

### Hospital and patient characteristics

**Figure 1:**
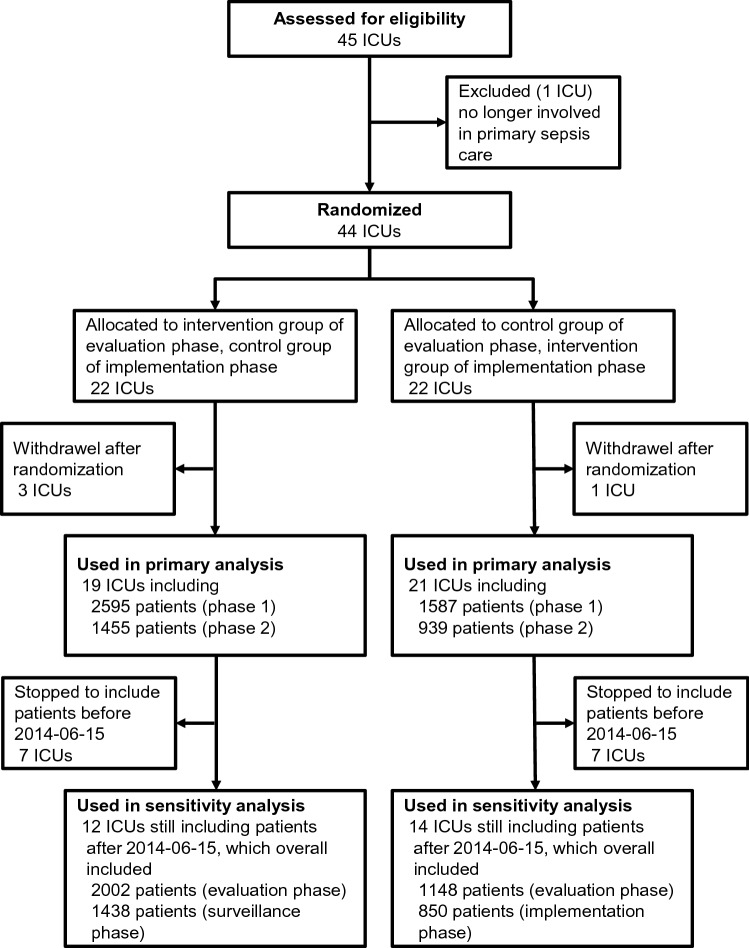
Inclusion of hospitals and patients.

Figure 1 shows the CONSORT flow diagram. Intervention hospitals included less patients than control hospitals across both study phases (N = 2526 vs. N = 4050). Because of a decrease of participating hospitals during the course of the trial, numbers of included patients decreased in both groups between phases (intervention: N = 1587 vs. N = 939; control: N = 2595 vs. N = 1455, Supplementary Fig. S1). Of the 40 initial hospitals, 26 continued to include patients until the second half of the evaluation phase. Hospital characteristics were nearly equally distributed between intervention and control group (Supplementary Table S1).

Table 1 compares demographic and clinical characteristics of patients across phases between groups. There were no marked differences between groups or between phases.

**Table 1:** Demographic and clinical characteristics of patients.

		All patients	Intervention group		Control group		p-value of test of difference-in-differences^b^
	N of cases with available data (intervention group control group)	N = 6576	Phase 1, N = 1587	Phase 2, N = 939	Phase 1,N = 2595	Phase 2, N = 1455	
Age	2525/2526, 4050/4050	70 [59, 77]	70 [59, 77]	71 [60, 78]	70 [59, 77]	70 [58, 77]	0.163
Sex: male	2526/2526, 4050/4050	4094 (62.3)	1011 (63.7)	579 (61.7)	1600 (61.7)	904 (62.1)	0.487
Origin of infection: community acquired	2526/2526, 4049/4050	2976 (45.3)	740 (46.6)	493 (52.5)	1078 (41.6)	665 (45.7)	≤ 0.001
Nosocomial (ICU/IMC)		1481 (22.5)	415 (26.1)	159 (16.9)	580 (22.4)	327 (22.5)	
Nosocomial (general ward)		2118 (32.2)	432 (27.2)	287 (30.6)	936 (36.1)	463 (31.8)	
Location at onset of sepsis: ICU	2526/2526, 4050/4050	3325 (50.6)	915 (57.7)	391 (41.6)	1356 (52.3)	663 (45.6)	≤ 0.001
Emergency room		1042 (15.8)	205 (12.9)	182 (19.4)	376 (14.5)	279 (19.2)	
Operating room		696 (10.6)	115 (7.2)	93 (9.9)	309 (11.9)	179 (12.3)	
General ward		906 (13.8)	189 (11.9)	156 (16.6)	356 (13.7)	205 (14.1)	
Emergency physician		218 (3.3)	27 (1.7)	57 (6.1)	71 (2.7)	63 (4.3)	
IMC		389 (5.9)	136 (8.6)	60 (6.4)	127 (4.9)	66 (4.5)	
Focus of infection: respiratory	2524/2526, 4049/4050	2688 (40.9)	648 (40.9)	338 (36)	1068 (41.2)	634 (43.6)	0.684
Focus of infection: abdominal	2524/2526, 4049/4050	2439 (37.1)	568 (35.8)	388 (41.3)	973 (37.5)	510 (35.1)	0.079
Focus of infection: urogenital	2524/2526, 4049/4050	876 (13.3)	216 (13.6)	169 (18)	314 (12.1)	177 (12.2)	0.763
Focus of infection: bones/soft tissue/wound	2524/2526, 4049/4050	724 (11)	171 (10.8)	85 (9.1)	291 (11.2)	177 (12.2)	0.01
Focus of infection: other/unknown	2524/2526, 4049/4050	878 (13.4)	232 (14.6)	110 (11.7)	343 (13.2)	193 (13.3)	0.143
Vasopressor use within 12 h after first organ dysfunction	2515/2526, 4049/4050	4930 (75.1)	1231 (78.1)	668 (71.1)	1950 (75.1)	1081 (74.3)	0.08
SAPS-II^a^	2155/2526, 3674/4050	48 [38, 60]	46 [36, 59]	47 [37, 56]	50 [39, 62]	47 [38, 58]	0.057
Lactate mmol/l^a^	2422/2526, 3928/4050	2.6 [1.6, 4.8]	2.4 [1.56, 4.3]	2.52 [1.58, 4.62]	2.8 [1.6, 5.2]	2.6 [1.5, 4.77]	0.014
Platelet count^a^	2511/2526, 4028/4050	191 [120, 288]	186 [118, 279.5]	188 [121, 293.25]	192 [119, 288]	196 [124, 292]	0.974
Base excess^a^	2441/2526, 3923/4050	−3.5 [−7.8, 2.3]	−2.2 [−6, 3.3]	−2.9 [−7.4, 2.6]	−4 [−8.3, 1.6]	−4.4 [−8.3, 1.4]	0.637

Descriptive statistics given as median [1st quartile, 3rd quartile] or N (%). All 40 hospitals included in the analyses. Data on phase 1 have been previously published^16^.

*ICU* intensive care unit, *IMC* intermediate care unit.

^a^Assessed as maximum value during the first 24 h after first infection-related organ dysfunction.

^b^Test of differences between groups regarding the change from phase 1 to phase 2. P-value obtained by testing the interaction effect between group and phase in a hierarchical generalized linear model with a random slope. For continuous variables a linear link-function was used, for dichotomous variables a logit-link was used, for categorical variables a multinomial model with a logit-link was used.

### Outcomes

**Figure 2:**
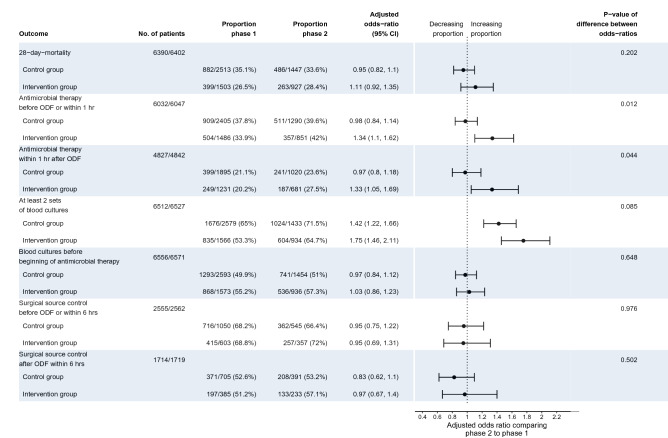
Difference-in-differences analysis of primary and secondary outcomes. Analyses based on data of 40 participating hospitals. Adjusted odds-ratios and p-values result from hierarchical generalized linear models with a logit link adjusted for the covariates age, sex, origin of infection, focus of infection, location at onset of infection and vasopressor use during the first 12 h. Difference-in-differences tested by an interaction effect between study phase and group (control vs. intervention). No. of patients gives the number of cases with complete data both on outcome and confounders compared to the total number of cases were the respective outcome was measured. Intraclass correlations (ICC): 28-day-mortality, ICC = 0.02; Antimicrobial therapy before ODF or within 1 h, ICC = 0.08; Antimicrobial therapy within 1 h after ODF, ICC = 0.04; At least 2 sets of blood cultures, ICC = 0.06; Blood cultures before beginning of antimicrobial therapy, ICC = 0.08; Surgical source control before ODF or within 6 h, ICC = 0.05; Surgical source control after ODF within 6 h, ICC = 0.03. ODF: Organ dysfunction. Data on phase 1 have been previously published^16^.

**Figure 3:**
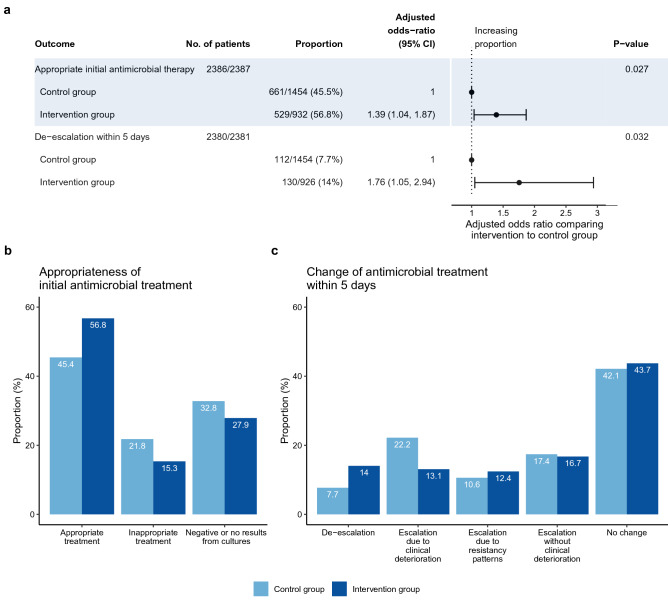
Comparison between groups during phase 2 of the trial regarding appropriateness and de-escalation of antimicrobial therapy. Analyses based on data of 29 participating hospitals. **(a)** Adjusted odds-ratios and p-values result from hierarchical generalized linear models with a logit link adjusted for the covariates age, sex, origin of infection, focus of infection, location at onset of infection and vasopressor use during the first 12 h. Since definitions of measures were changed between phases, no difference-in-difference analysis was possible. No. of patients gives the number of cases with complete data both on outcome and confounders compared to the total number of cases were the respective outcome was measured. Intraclass correlations (ICC): Appropriate initial antimicrobial therapy, ICC = 0.03; De-escalation within 5 days, ICC = 0.03. **(b)** Barplot on appropriateness of initial antimicrobial treatment. **(c)** Barplot on change of antimicrobial treatment within five days after sepsis onset.

Figure 2 presents results of the difference-in-differences analysis. There was no difference-in-differences regarding 28-day mortality between groups (adjusted OR comparing phase 2 to phase 1 of 0.95 [95% CI 0.82,1.1] for control group and 1.11 [0.92, 1.35] for intervention group, respectively; p-value of difference in odds ratios was 0.202). Among the included 6576 patients, 6047 (92%) had received a new antimicrobial treatment related to the infection, which caused sepsis. The intervention group showed an increase of proportion of antimicrobial therapy within the first hour from 33.9 to 42% (adjusted OR: 1.34 [1.1, 1.62]), while there was no change in the control group (adjusted OR: 0.98 [0.84, 1.14], significant difference between odds ratios with p = 0.012). This corresponds to a change in median time to antimicrobial therapy by 33 min from 120 min (1st quartile: 25 min, 3rd quartile: 360 min) to 87 min (10.5 min, 282.5 min) in the intervention group compared to no change in the control group (90 min [8 min, 300 min], and 90 min [11 min, 281 min], respectively). Proportion of taking at least two sets of blood cultures increased in both groups from phase 1 to phase 2 (OR = 1.42 [1.22, 1.66] and OR = 1.75 [1.46, 2.11] for control and intervention group respectively) with no significant difference between groups (p = 0.085). Neither the proportion of blood cultures taken before beginning of antimicrobial therapy nor the proportion of conducting surgical source control within 6 h changed significantly between phases for any group. Compared to the control group during phase 2, the intervention group showed a higher proportion of appropriate initial antimicrobial therapy (56.8% vs. 45.5%, p = 0.027) and a higher proportion of de-escalation within 5 days (14% vs. 7.7%, p = 0.032, Fig. 3). Including only study centers participating until the second half of phase 2 did not alter the pattern of results except for increased p-values due to reduced sample size (Supplementary Figs. S2 and S3).

Supplementary Figure S4 presents the quarterly course of outcomes during the trial. The proportion of antimicrobial therapy within 1 h increased in the intervention group during the first year of the implementation phase with a tendency to drop again in the second year. Proportion of having taken at least two sets of blood cultures improved quickly and remained high both in control hospitals during phase 1 and intervention hospitals during phase 2. Proportion of appropriate initial antimicrobial therapy and proportion of de-escalation within 5 days were consistently higher during phase 2 in intervention compared to control hospitals.

### Results of process evaluation

Table 2 shows topics discussed during educational sessions of study coordinators with QI teams as well as aims set in these meetings. The meetings had a great focus on building and improving the structures and processes for quality management (e.g., improving the quality of data documentation, involving additional departments in the QI process, improving meeting-frequency, staffing and coordination of the QI team). Regarding actual quality improvement activities, staff education, management of blood cultures and antimicrobials, and in-depth analysis of problems of care were most prominent. The improvement of surgical focus control was never documented as a topic of discussion or aim. The documentation of meetings was not suitable to analyze the degree of achieving the aims set.

**Table 2:** Contents of the educational sessions of study coordinators with quality improvement teams.

Topics and aims	Number of centers (total N = 17)
**Topics discussed during educational sessions with QI teams**	
Improving management of blood cultures	13
Discussing individual results of the quality report	14
Discussing the lacking quality of the documented data for quality reporting	11
Identifying structural barriers to treatment of sepsis, which is adherent to guidelines	12
Discussing, how additional departments could be involved in the QI process	7
Discussing, how to improve antimicrobial treatment according to guidelines	5
Discussing the success of implemented measures for quality improvement	5
Discussing how to plan and conduct education on sepsis for hospital staff	3
Discussing the status and improvement of staffing of the QI team	3
**Aims set during the educational sessions**	
Conduction of education on sepsis for hospital staff	13
Conduction of focus group interviews with clinical staff to identify problems of care	11
Improving blood culture management	11
Improving quality of data documented for quality reporting	11
Making antibiotics readily available on wards	8
Developing a standard operating procedure for management of sepsis	9
Having more meetings of the QI team	8
Distribution of educational material (posters, flyers) among clinical staff	7
Involving additional departments in the QI team and QI process	6
Implementing regular case conferences on cases with sepsis	6
Making educational material available in the intranet	5
Developing a sepsis screening checklist	5
Recruiting more members for the QI team	4
Improving coordination of tasks within the QI team	3

Results of qualitative analyses of open-ended questions distributed to study coordinators after each educational session with quality improvement (QI) teams of intervention hospitals during implementation phase. Data were available for 17 QI teams. Every category was only counted once per QI team. Only categories present among at least three QI teams are shown.

Table 3 presents barriers to implementation of QI as perceived by study coordinators and QI team members. The reported barriers showed overlap indicating (a) insufficient resources in time and manpower both in the QI team as well as among hospital staff in general, (b) insufficient involvement of departments outside the ICU and of nurses in the QI process, and (c) reduced quality of audit and feedback because of low and infrequent inclusion of cases by the hospitals.

**Table 3:** Barriers to implementation of quality improvement as perceived by quality improvement teams and study coordinators.

Categories derived from qualitative analyses	Number of QI teams where issue was perceived by QI team members (total n = 14)	Number of QI teams where issue was perceived by study coordinators (total n = 17)
Shortage of time of QI team members	12	–
Lack of motivation of QI team members	7	–
Shortage of manpower within the QI team	5	3
Relevant hospital departments not represented in the QI team	5	7
High staff turnover in relevant hospital departments	4	–
Lack of leadership support (department or hospital)	4	–
Low and infrequent documentation of cases for audit and feedback	3	7
Heavy workload of QI team members	3	–
Rare QI team meetings	3	–
No nurses included in QI team	–	4
Conflicts within the QI team	–	3
Too strong hierarchy in the QI team	–	3
Unstructured working process of the QI team	–	3

Results of qualitative analyses of open-ended questions distributed to study coordinators after each educational session with quality improvement (QI) teams of intervention hospitals during implementation phase and of open ended questions distributed to QI teams of intervention hospitals during the second half of the implementation phase. Data by QI teams were available for 14 QI teams, data by study coordinators were available for 17 QI teams. Every category was only counted once per QI team per data source. Only categories present among at least three QI teams are shown.

## Discussion

In our study, hospitals receiving the educational intervention did not show a decrease in patients’ mortality compared to hospitals receiving only feedback of quality indicators. The intervention was associated with an increase in the proportion of patients receiving antimicrobial therapy within the first hour after onset of sepsis. Hospitals in the intervention group also showed higher rates of appropriate initial antimicrobial therapy and appropriate de-escalation within 5 days.

There are several possible reasons why the improved intervention showed more effects on care processes than the initial intervention^16^. First, there was a more structured approach to the educational outreach. Moreover, more efforts were made to include departments outside of the ICU as well as nurses in the QI teams. Feedback, active reminders and educational outreach included additional emphasis on adequacy of initial antimicrobial therapy and early de-escalation. Changes in time to antimicrobial therapy were observed in intervention hospitals only, proportions of adequate initial antimicrobial therapy and de-escalation were higher in this group. Control hospitals received the same quality reports and active reminders as intervention hospitals in phase 2 but did not receive educational meetings with study coordinators. This might indicate that the regular support by external experts might have additional effects beyond a simple performance feedback. Because of its randomized controlled design, our study lends strength to the evidence, that sepsis care processes can actually be improved by educational interventions.

Despite improvements in antibiotic treatment, no decrease of mortality in patients with sepsis was observed. A systematic review and meta-analysis on studies to increase implementation of sepsis bundles found a large average effect on mortality with an odds ratio of 0.66^11^. None of the included 48 studies used a controlled design. Uncontrolled before-after-designs are prone to overestimation of intervention effects due to secular trends, regression to the mean, or stage migration^14^. Likewise, a meta-analysis on studies for implementation of care bundles found larger treatment effects among non-randomized compared to randomized trials^25^. Two recent multi-centre studies using a time-series analysis confirmed our findings as they did not show an improvement of survival although time to antimicrobial therapy was decreased^26,27^. Planning of the MEDUSA trial relied on results of an early retrospective observational study showing an increase of 7% per hour delay in beginning of antimicrobial therapy^28^. However, more recent large observational studies showed significant but relatively small effects on mortality between 0.3 and 1.4% increase per hour^7,29–31^. We found an increase in mortality of 0.4% per hour delay in the MEDUSA data^32^. Thus, the observed reduction in the intervention group in time to antimicrobial therapy of half an hour would be only associated with an expected 0.2% reduction in mortality. Ferrer et al. estimated that 50,000 patients would be necessary to test for an effect on mortality that could be caused by half an hour reduction of time to antimicrobial therapy^26^. Therefore, it seems necessary to include additional or all elements of the sepsis six bundles to achieve substantial reductions in mortality^3,4,11,12,33–36^.

In our study, improvements were achieved in management of blood cultures as well as in timeliness and appropriateness of antimicrobial therapy. Analyses of topics and aims discussed in educational outreach sessions showed that there was a great emphasis on improvements in these areas. The large effects on the number of blood cultures observed in both groups of hospitals, might partly be explained by the introduction of packs, which bundled three blood culture sets in part of the participating hospitals. At the same time, the process documentation reports no efforts to improve timeliness of source control. Improvement of implementation of sepsis guideline recommendations is a complex endeavor—requiring coordination and cooperation by a multiprofessional and multidisciplinary team to conduct a sequence of interdependent tasks, often involving several departments^37,38^. This is especially true, if the coordination of the operating room needs to be changed to achieve timelier source control for patients with sepsis. Since a large part of the work of QI teams was building and improving structures and processes for quality management—such as involving additional departments and organizing regular meetings—they might have focused the existing resources on “reaching for the lower hanging fruits”.

Process evaluation by surveying local quality improvement teams as well as visiting study coordinators revealed lacking time and manpower as well as lacking support by management and departments outside the ICU to successfully implement changes, and partly even lacking resources to accomplish timely and complete documentation of cases. In Germany, hospitals invest many resources in legally mandated quality assurance activities. At the same time, our results implicate that scarce resources for additional voluntary quality initiatives hinder improvements of sepsis care. Some governmental public bodies, like those of the state of New York, of Ireland, and of Norway, started mandatory programs for hospitals to improve sepsis care that were able to increase implementation of bundle elements and to decrease mortality^33,34,39,40^. In Germany, improvements of sepsis care might be fostered by a mandatory sepsis quality assurance program, as is currently in development under the responsible governmental agency.

Singer and colleagues recently^41^ questioned the benefits of quality initiatives to decrease the time to initial antimicrobial therapy mainly because of the risk of increasing antimicrobial resistance. In our trial, the intervention group showed higher rates of appropriate initial antimicrobial therapy as well as de-escalation of antimicrobial therapy within 5 days and also decreased delays to initial antimicrobial therapy compared to baseline. Quality initiatives on improvement of sepsis guidelines implementation should always incorporate elements aimed at monitoring and decreasing inappropriate use of antimicrobials to prevent negative side effects and increased resistance^42^.

This trial is one of the few evaluations of a quality initiative on sepsis care using a controlled design, and one of only two studies using randomization^11,34,43^. Cluster-randomized trials are more prone to allocation bias due to randomization failure, because numbers of clusters are often quite small^44^. The analysis of the first intervention phase of the MEDUSA trial was biased by such a randomization failure^16^. By using the first intervention phase as a baseline, a difference-in-differences design was now applied that most likely increased test-power^45^ and the validity of conclusions by comparing the development in outcomes across time between groups^14^. Further strengths of this study in comparison to the first intervention phase^16^ are an improved intervention strategy, which resulted in improved timeliness of antimicrobial therapy; emphasis on appropriateness of antimicrobial therapy, which might have led to increased rates of appropriate treatment and de-escalation; an extended process evaluation based on documentation of the educational outreach sessions and surveys of study coordinators and local QI teams, which allowed more detailed insight on implementation activities and barriers to success. Our study also has limitations. Since the control group had continued to receive elements of the intervention also in phase 2 of the study, the difference-in-differences analyses might have underestimated the effects, which could have been achieved by the intervention. Because endpoints on adequacy and de-escalation of antimicrobial therapy were newly defined for the second intervention phase, no baseline was available and the difference between groups might be biased by randomization failure. The drop-out of hospitals during the study might have biased results, if hospitals with worse performance would have shown a higher tendency for drop out. To control this bias, we conducted a sensitivity analysis including only hospitals that took part in the whole study which showed the same pattern of results. Including only patients with sepsis treated on ICU is another bias. Therefore, it was not possible to detect benefits for patients treated only on normal wards. Our intervention provided support to participating hospitals to implement a quality management for sepsis. However, QI teams were completely self-responsible on defining concrete aims, resources, and solutions. A more standardized approach with concrete criteria and milestones to achieve in the given time might have been more successful. Finally, the study did not include measures on possible negative side effects such as increased inappropriate use of antimicrobials among patients without infections.

## Conclusions

The findings from this pragmatic cluster-randomized controlled trial confirm the possibility to achieve improvements in processes of sepsis care by multifaceted educational interventions. To achieve reductions of mortality, it is necessary to include additional bundle elements of sepsis guidelines to quality programs beside anti-infections measures. Results of the process evaluation indicate that many hospitals lack resources to achieve critical improvements in their quality of sepsis care. Additional financial support for quality management activities as well as governmental mandated quality control programs could be a way to improve care for patients with sepsis in Germany.

## Supplementary Information

Supplementary Information.

## Data Availability

All data requests should be submitted to Dr. Bloos (frank.bloos@med.uni-jena.de) for consideration. Access to the anonymised data might be granted following review by the Center for Sepsis Control and Care (CSCC) and the Jena University Hospital.
