# Supplementary material for: A multifaceted educational intervention improved anti-infectious measures but had no effect on mortality in patients with severe sepsis
Source: Sci Rep. 2022 Mar 10;12:3925. doi: 10.1038/s41598-022-07915-9 (PMC8913650; doi:10.1038/s41598-022-07915-9)
Supplement: Supplementary file 1 — Supplementary Information. [file 41598_2022_7915_MOESM1_ESM.docx]

A multifaceted educational intervention improved anti-infectious measures but had no effect on mortality in patients with severe sepsis

Daniel Schwarzkopf, Claudia-Tanja Matthaeus-Kraemer, Daniel O. Thomas-Rüddel, Hendrik Rüddel, Bernhard Poidinger, Friedhelm Bach, Herwig Gerlach, Matthias Gründling, Matthias Lindner, Christian Scheer, Philipp Simon, Manfred Weiss, Konrad Reinhart, and Frank Bloos for the MEDUSA study group.

Supplementary material

Supplementary methods

Description of the multifaceted intervention

The description of the original intervention is cited from Bloos et al 2017 [1].

**Formation of local quality improvement teams.** Local quality improvement teams consisted of experienced ICU physicians and nurses guided by a local champion. Team members were required to have knowledge and expertise in ICU processes, needed to be accepted representative of their departments, had to be authorized to make decisions, and to have support of the management. Additional members were recruited at the discretion of the local team according to hospital size and chosen implementation strategies. Multidisciplinarity and multiprofessionality of the team were strongly encouraged by the study coordinators. The teams were asked to hold frequent meetings, i. to review the key measures of guideline compliance regularly distributed by the study coordinators, ii. to initiate and maintain change strategies agreed upon during the educational outreach visits, and, iii. to distribute the related information to the hospital staff.

Changes made for the evaluation phase: The composition of the quality improvement teams was more thoroughly monitored by the study coordinators and documented during educational outreach sessions. Encouragement to include additional relevant departments was repeatedly given during the educational outreach sessions.

**Educational outreach.** The study coordinators conducted visits to each quality improvement team up to three times a year. These meetings served to train the quality improvement team in change management (i.e., process analysis, focus group discussion), discuss the current benchmarks, and setup specific goals for the quality improvement team. Main focus of the training was to improve sepsis recognition by the medical staff and shorten time to primary care specifically addressing time to antimicrobial therapy (AT).

*Changes made for the evaluation phase:* Outreach sessions were now conducted by at least two study coordinators (physicians, social scientists) to enhance moderation and documentation of the sessions. Documents were developed for a structured planning and documentation of each session. Each session was structured to assess goal attainment for aims set in previous sessions, interpret and discuss individual quality indicators, to formulate and document aims and responsibilities within the quality improvement teams for the time after the session. Additionally, centers were offered focus group interviews on barriers to early detection and treatment of sepsis. These interviews were conducted involving nurses and physicians from emergency departments, normal wards, intermediate care and intensive care units. In total, five focus group interviews were conducted among hospitals of the intervention group and results were reported to the local quality improvement teams. Details on methods and results of focus group interviews are presented elsewhere [2].

**Audit and feedback.** Key measures of guideline compliance such as time to AT, time to source control, and compliance with appropriate taking of blood cultures, along with patient outcomes (including mortality, ICU and hospital length of stay) were summarized into individual reports allowing blinded comparison with the other study centers.

*Changes made for the evaluation phase:* Additional quality indicators were included in the report to foster early de-escalation of AT among patients with sepsis. These included reasons for de-escalation or escalation of AT (microbiological results, clinical reasoning), and adequacy of initial AT.

**Active reminders.** Monthly e-mail alerts reported outliers with time to AT of more than two hours.

*Changes made for the evaluation phase:* The monthly reports were enhanced by including additional information on pathogens identified by microbiological diagnostics, type of AT medication used, and reasons for escalation or de-escalation of AT.

**Passive reminders.** The quality improvement teams received educational materials to increase vigilance regarding diagnosis and therapy of sepsis and attract attention for the project (pocket cards, flyers, posters).

*Changes made for the evaluation phase:* Content of educational material was updated to correspond to newest scientific evidence.

Supplementary figures


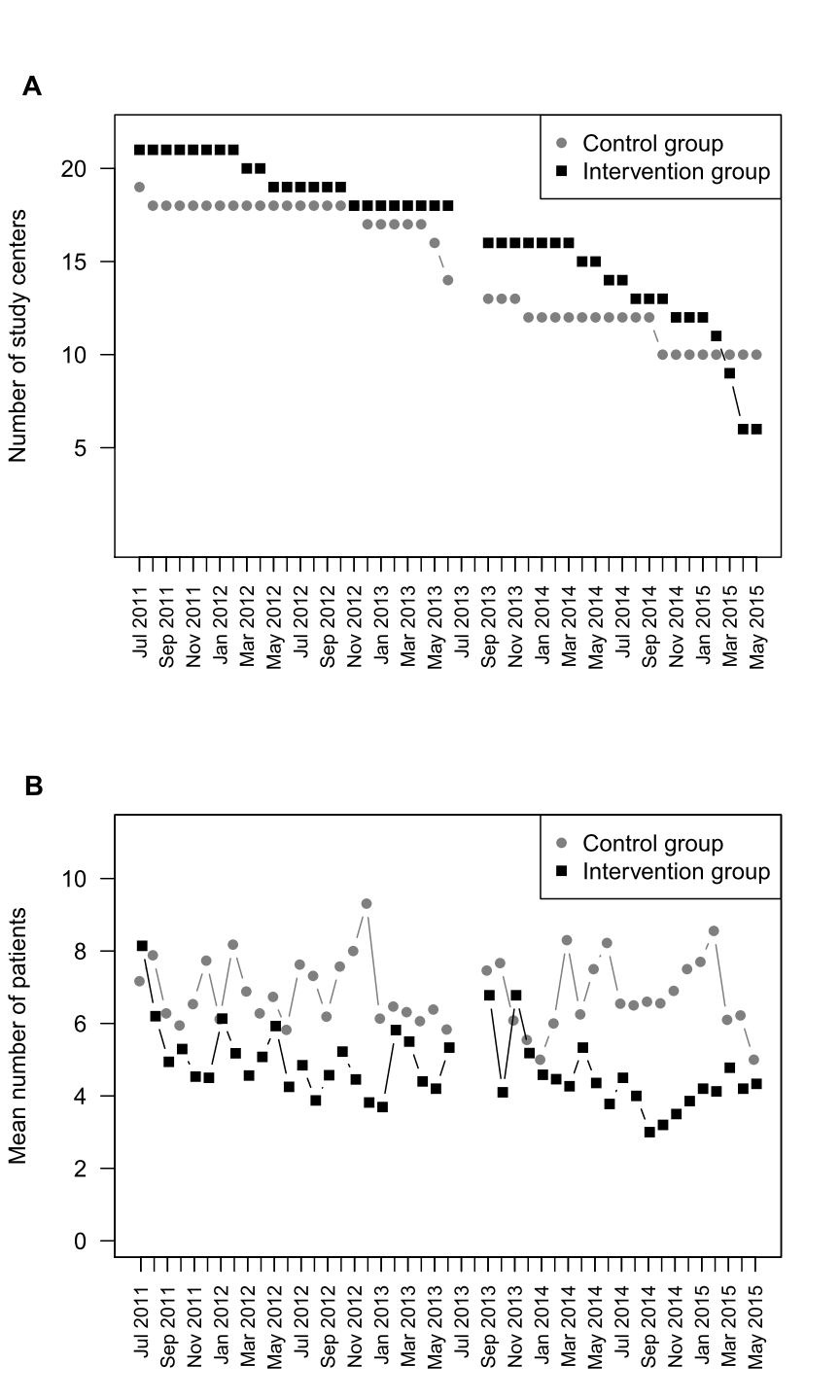


**Supplementary Figure S1 a.** Number of centers in each group, which continued to include patients after the respective month.

**Supplementary Figure S1 b.** Mean number of patients included per center and month.


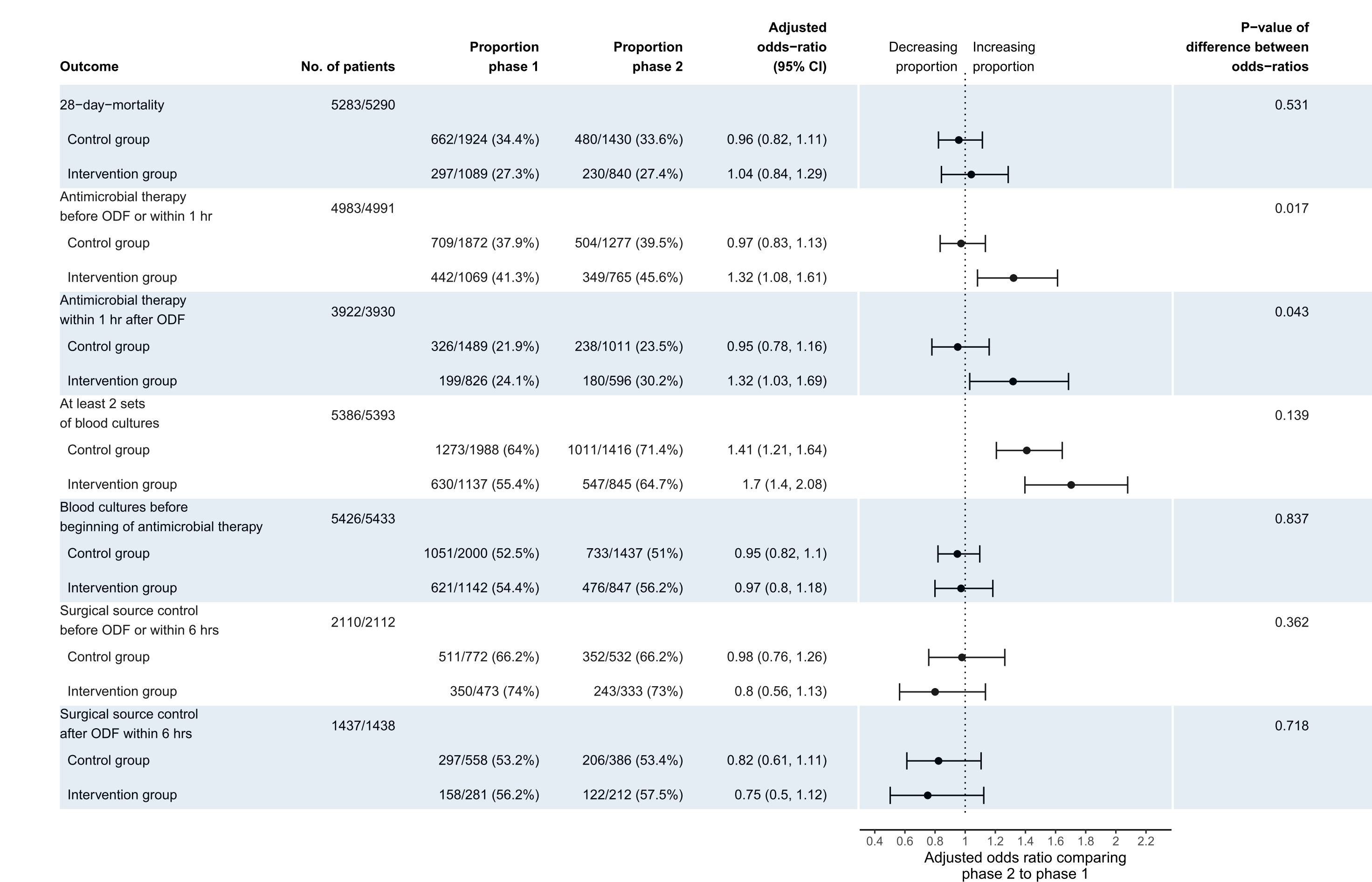


**Supplementary Figure S2: Difference-in-differences analysis of primary and secondary outcomes in subgroup of study centers participating until the second half of phase 2.** Analyses based on data of 26 participating hospitals. Adjusted odds-ratios and p-values result from generalized hierarchical linear models with a logit link adjusted for the covariates age, sex, origin of infection, focus of infection, location at onset of infection and vasopressor use during the first 12 h. Difference-in-differences tested by an interaction effect between study phase and group (control vs. intervention). No. of patients gives the number of cases with complete data both on outcome and confounders compared to the total number of cases were the respective outcome was measured. Intraclass correlations (ICC): 28-day-mortality, ICC = 0.02; Antimicrobial therapy before ODF or within 1 hr, ICC = 0.05; Antimicrobial therapy within 1 hr after ODF, ICC = 0.03; At least 2 sets of blood cultures, ICC = 0.06; Blood cultures before beginning of antimicrobial therapy, ICC = 0.07; Surgical source control before ODF or within 6 hrs, ICC = 0.04; Surgical source control after ODF within 6 hrs, ICC = 0.02. ODF: Organ dysfunction.


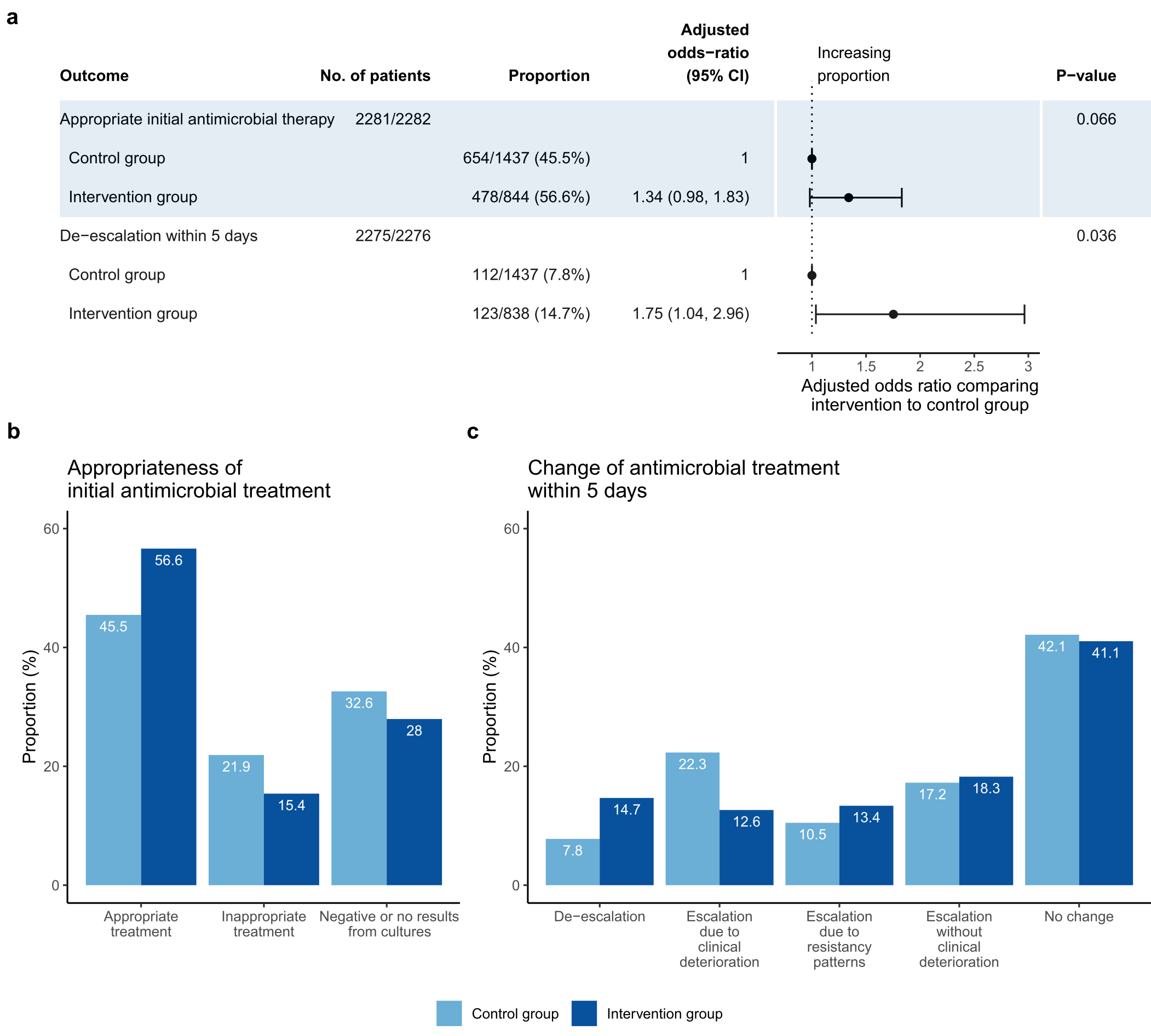


**Supplementary Figure S3: Comparison between groups during phase 2 of the trial only including subset of study centers participating until the second half of the intervention phase.** Analyses based on data of 26 participating hospitals. Since definitions of measures were changed between phases, no difference-in-difference analysis was possible. Panel a: Adjusted odds-ratios and p-values result from generalized hierarchical linear models with a logit link adjusted for the covariates age, sex, origin of infection, focus of infection, location at onset of infection and vasopressor use during the first 12 h. No. of patients gives the number of cases with complete data both on outcome and confounders compared to the total number of cases were the respective outcome was measured. Intraclass correlations (ICC): Appropriate initial antimicrobial therapy, ICC = 0.04; De-escalation within 5 days, ICC = 0.04. Panel b: Barplot on appropriateness of initial antimicrobial treatment. Panel c: Barplot on change of antimicrobial treatment within five days after sepsis onset.


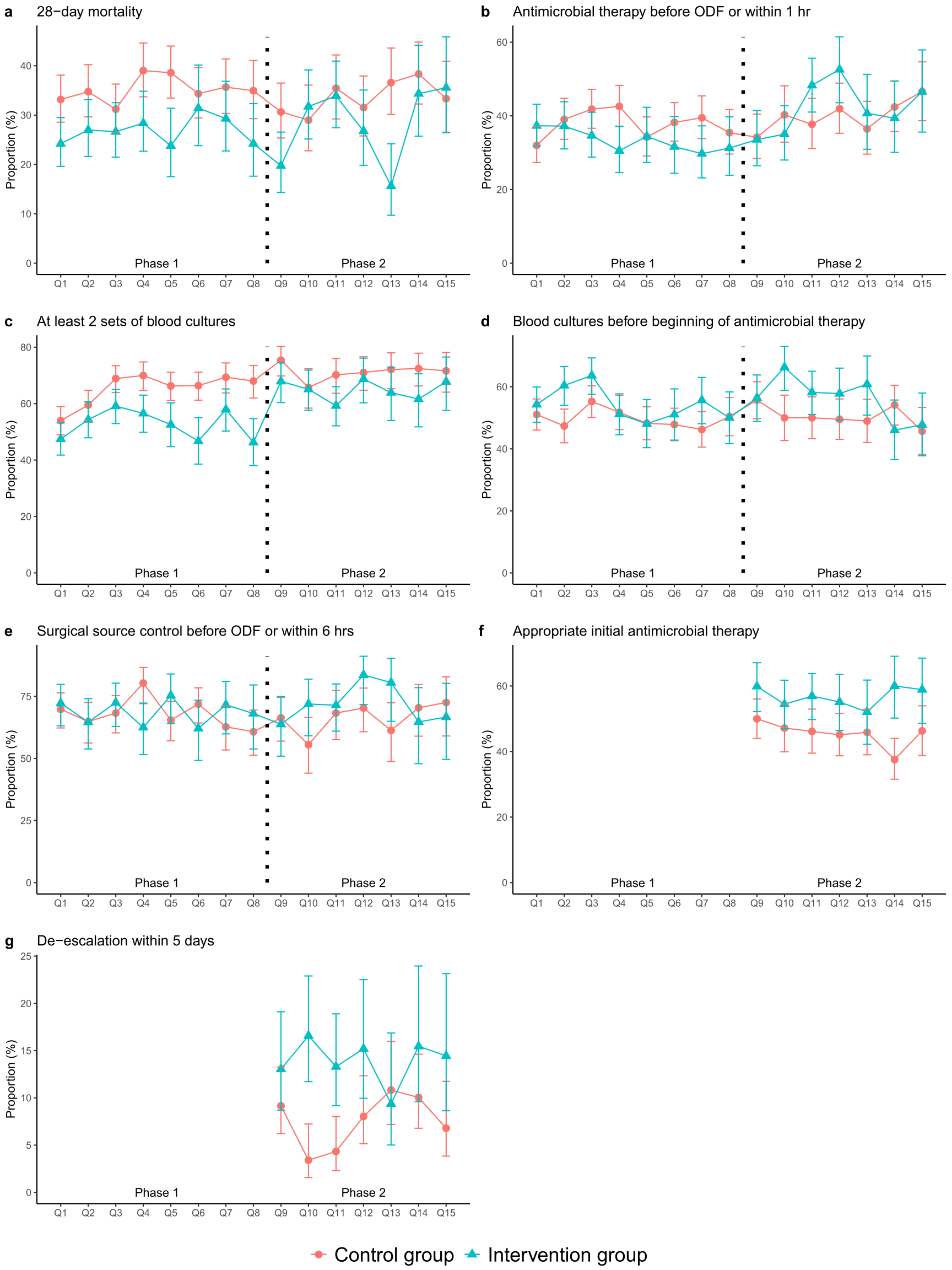


**Supplementary Figure S4.** Course of primary and secondary outcomes during the trial. Error bars give the 95% confidence limit.

Supplementary tables

**Supplementary Table S1.** Characteristics of participating hospitals

|  | Group 1 (intervention in surveillance phase, control in trial phase)  n=19 | Group 2 (control in trial phase, intervention in surveillance phase)  n=21 |
| --- | --- | --- |
| *Hospital operator*  public  private  non-profit | 11 (57.9%)  6 (31.6%)  2 (10.5%) | 12 (57.1%)  6 (28.6%)  3 (14.3%) |
| *Level of care*  Primary  Secondary  Tertiary | 5 (26.3%)  7 (36.8%)  7 (36.8%) | 7 (33.3%)  5 (23.8%)  9 (42.9%) |
| ICU beds, median (IQR) | 14 [11, 24] | 20 [10, 38] |
| Hospital beds, median (IQR) | 569 [440, 1050] | 669 [418, 1009] |
| Inhospital microbiology department | 13 (68.4%) | 15 (71.4%) |

Descriptive statistics given as N (%) or median [1^st^ quartile, 3^rd^ quartile].

Supplementary Literature

1. Bloos F, Rüddel H, Thomas-Rüddel D, Schwarzkopf D, Pausch C, Harbarth S, Schreiber T, Gründling M, Marshall J, Simon P *et al*: **Effect of a multifaceted educational intervention for anti-infectious measures on sepsis mortality: a cluster randomized trial**. *Intensive Care Med* 2017, **43**(11):1602 - 1612.

2. Matthaeus-Kraemer CT, Thomas-Rueddel DO, Schwarzkopf D, Rueddel H, Poidinger B, Reinhart K, Bloos F: **Crossing the handover chasm: Clinicians' perceptions of barriers to the early detection and timely management of severe sepsis and septic shock**. *J Crit Care* 2016, **36**:85-91.
